# Bibliometric analysis of trends, innovations, and the future of CBT-based mobile interventions for depression

**DOI:** 10.3389/fmed.2025.1710291

**Published:** 2025-12-17

**Authors:** Zhe Gao, Tingzhou Zhao, Yuhang Li, Wenhao Huang, Junxi Tang, Xujun Yu, Yulin Li, Tangming Peng

**Affiliations:** 1 School of Medicine and Life Sciences, Chengdu University of Traditional Chinese Medicine, Chengdu, China; 2 Hospital of Chengdu University of Traditional Chinese Medicine, Chengdu University of Traditional Chinese Medicine, Chengdu, China

**Keywords:** bibliometric analysis, citespace, cognitive behavioral therapy, depression, mobile mental health applications

## Abstract

**Background:**

Depression is a major global health challenge, and traditional cognitive behavioral therapy (CBT) is constrained by therapist shortages and economic barriers. CBT-based mobile applications offer a scalable and accessible alternative, yet a comprehensive overview of their global research landscape remains limited.

**Objective:**

To map global research trends, clinical progress, and emerging frontiers in CBT-based mobile applications for depression using bibliometric methods.

**Methods:**

Relevant studies were systematically retrieved from the Web of Science Core Collection, PubMed, and PsycINFO from inception to June 25, 2025. CiteSpace 6.4 R1, Microsoft Excel 2019, and Python were used for visualization and data analysis, including temporal publication trends, co-authorship, co-citation, keyword analyses, and citation burst detection.

**Results:**

The WoSCC analysis identified 350 articles published between 2013 and 2025, showing a marked growth trajectory with leading contributions from the United States and major academic centers. Dominant themes included smartphone interventions, blended treatments, CBT for insomnia, and adolescent depression, while citation bursts indicated recent shifts toward prevention, technology integration, and standardized outcome measures. The PubMed analysis included 72 clinical trial articles, highlighting randomized controlled trials as the predominant design and revealing growing interest in integrating interpersonal therapy and mindfulness within broader, interdisciplinary treatment frameworks. The PsycINFO analysis comprised 20 articles and provided a complementary behavioral science perspective, emphasizing mobile phone–delivered CBT for major depression, digital interventions targeting comorbid social anxiety, culturally adapted applications for Chinese cultural groups, and emerging work linking mobile health and virtual reality.

**Conclusions:**

Research on CBT-based mobile applications for depression is rapidly advancing toward more personalized, adaptive, and preventive digital interventions grounded in robust clinical and behavioral evidence. Strengthening global, interdisciplinary collaboration and leveraging innovative technologies will be critical for translating these tools into effective, scalable services. Over the next 5–10 years, key research streams are likely to include the integration of Artificial Intelligence (AI) and large language models (LLMs) into mobile CBT platforms and the convergence of app-based interventions with sensor-based digital phenotyping, wearable devices, and immersive technologies to enhance real-time monitoring, user engagement, and long-term outcomes, with the potential to narrow treatment gaps across diverse populations.

**Systematic review registration:**

https://osf.io/, Identifier: https://doi.org/10.17605/OSF.IO/YCSR8.

## 1 Introduction

Depression is one of the most prevalent psychiatric disorders worldwide, characterized by persistent low mood or loss of interest or pleasure in activities (1–3). It imposes a substantial global public health burden. According to data from the Global Burden of Disease Study 2021 (GBD 2021), an estimated 332.41 million individuals worldwide were affected by depression in 2021 (4). Consequently, developing effective, accessible, and scalable treatments for depression has become critically important.

Cognitive Behavioral Therapy (CBT) has been widely acknowledged as a highly effective psychological intervention for depression (5), consistently demonstrating robust efficacy across diverse patient populations. Despite substantial empirical support for CBT, its widespread implementation faces numerous practical challenges, including a severe shortage of qualified therapists, significant economic burden, and high treatment drop-out rates (6, 7). Recent advancements in psychological interventions have expanded CBT by integrating mindfulness and acceptance-based approaches, resulting in the emergence of Mindfulness-Based Cognitive Therapy (MBCT) (8) and Acceptance and Commitment Therapy (ACT) (9).

In parallel, the rapid development of mobile technology has offered promising solutions to address these practical limitations. Particularly, mobile mental health applications grounded in CBT principles have garnered significant attention due to their potential advantages, including cost-effectiveness, extensive reach, anonymity, and flexibility of use. In this study, the term intervention refers specifically to the therapeutic use of these mobile technologies as structured vehicles for delivering CBT based strategies to individuals with depression, so that digital platforms and cognitive behavioral methods operate as an integrated treatment package rather than as separate elements. For instance, when CBT techniques such as behavioral activation, cognitive restructuring, and skills training are embedded within smartphone applications or other mobile interfaces and used to guide patients' day-to-day actions and reflections, the combined system constitutes the intervention under investigation. Within this framework, intervention is therefore inseparable from both mobile delivery and CBT content, and denotes the concrete, technology enabled therapeutic processes that link evidence based psychological mechanisms to real-world clinical practice.

Although numerous studies have evaluated the efficacy and effectiveness of CBT-based mobile applications for managing depression, systematic analyses summarizing global research trends, research hotspots, and collaborative networks within this domain remain scarce. Bibliometric analysis is an effective approach to quantitatively assess and visually represent trends, collaboration patterns, and research hotspots within a specific academic field (10). CiteSpace, a widely utilized bibliometric analysis software tool (11), allows researchers to visualize research progress, identify emerging topics, and thereby facilitate comprehensive insights into a given research area.

This study therefore employs bibliometric analysis using CiteSpace to systematically map the global research trends and knowledge structures of CBT-based mobile applications for depression. To construct a truly comprehensive and multilayered map of this rapidly evolving domain, our research adopts an integrated three-database strategy. We first utilize the Web of Science Core Collection, the established standard for scientometric research, to build a macro-level knowledge map that provides a panoramic view of the intellectual landscape and reveals the foundational theories, broad research themes, and overall collaboration patterns that define the field. To specifically chart the course of clinical translation, where scientific insights are put into practice, we complement this with a micro-level analysis of a curated dataset of clinical trials from PubMed that traces how app-based CBT is evaluated in randomized designs and real-world patient populations. In addition, we incorporate PsycINFO as a specialized behavioral science and mental health database in order to capture core psychological and transdiagnostic work on mobile CBT that may not be comprehensively indexed in WoSCC or PubMed, including studies focused on comorbid social anxiety, cultural adaptations, and behavioral activation-driven interventions. Taken together, these three sources allow us to link macro-level publication structures with clinical endpoints and behaviorally grounded, culturally sensitive research, thereby offering a more nuanced and clinically relevant map of how CBT-based mobile interventions for depression are developing worldwide.

Crucially, rather than merging these distinct datasets—a process that would dilute and obscure the unique signals within intervention-based research—we analyze them in parallel. This deliberate methodological separation is a key strength of our study. It enables a comparative examination of the general scientometric corpus drawn from the Web of Science Core Collection, the behavioral and psychological corpus drawn from PsycINFO, and the specialized clinical trial corpus drawn from PubMed, and it provides a clear basis for how we use bibliometric evidence to characterize trends, innovations, and future directions in this field. In this context, trends refer to temporal and structural patterns in publication volume, collaboration networks, and thematic emphases that emerge across coauthorship, co-citation, and keyword maps, revealing how attention to particular populations, mechanisms, and technologies has evolved over time. Innovations denote emergent topics, methodological shifts, and technological or clinical developments that depart from previously dominant configurations, as indicated by newly formed or rapidly strengthening nodes, clusters, and citation bursts in these networks. The future is defined not as a distant or speculative horizon but as the short- to medium-term research priorities and implementation pathways most strongly implied by these recent trends and innovations, especially where signals from the scientometric and behavioral corpora converge with the trajectory of ongoing and planned clinical trials. By tracing how shifts in the broader academic and psychological literature are mirrored in the design, target populations, and outcome measures of CBT-based mobile applications for depression, this parallel analysis allows us to move beyond a static description of the literature and articulate a dynamic account of where the field is headed, identifying both synergies and potential gaps between foundational research and its practical application. In doing so, the study aims to provide clearer and more actionable insights for researchers, clinicians, and policymakers about how current trajectories in mobile CBT research are likely to shape the near future of digital mental health care.

## 2 WoSCC-based data and methods

### 2.1 WoSCC data source and search strategy

This study employed a multi-database bibliometric approach to comprehensively map the research landscape of CBT-based mobile applications for depression. The first phase involved a broad analysis of the Web of Science Core Collection to establish the overall intellectual structure of the field. The second phase involved a focused analysis of a curated dataset of clinical trials from PubMed to examine trends specifically within intervention-based research. A third phase consisted of a supplementary analysis of a behaviorally focused corpus retrieved from PsycINFO, a specialized database in psychology and mental health, in order to capture additional perspectives on CBT-based mobile interventions that may not be fully represented in Web of Science Core Collection or PubMed.

Relevant literature for the first phase of our analysis was retrieved from the Web of Science Core Collection, a comprehensive and authoritative global scientific database widely used for scientometric studies. The literature search covered the period from the inception of the database to June 25, 2025. To ensure comprehensive coverage, the search strategy included relevant terms, synonyms, alternative expressions, and truncation techniques. The complete, executable search query was: TS = ((“smartphone^*^” OR “mobile app^*^” OR “mobile application^*^” OR “smartphone application^*^” OR “mobile phone app^*^” OR “mental health app^*^”) AND (depress^*^ OR “major depressive disorder” OR MDD) AND (“cognitive behavioral therapy” OR “cognitive behavior therapy” OR CBT OR chatbot OR “conversational agent”)). The detailed search strategy is summarized in Table 1.

**Table 1:** Retrieval strategy for WoSCC.

**Set**	**Search query**
#1	TS = (“smartphone^*^” OR “mobile app^*^” OR “mobile application^*^” OR “smartphone application^*^” OR “mobile phone app^*^” OR “mental health app^*^”)
#2	TS = (depress^*^ OR “major depressive disorder” OR MDD)
#3	TS = (“cognitive behavioral therapy” OR “cognitive behavior therapy” OR CBT OR chatbot OR “conversational agent”)
#4	#1 AND #2 AND #3

### 2.2 Study selection and eligibility in WoSCC

**Figure 1:**
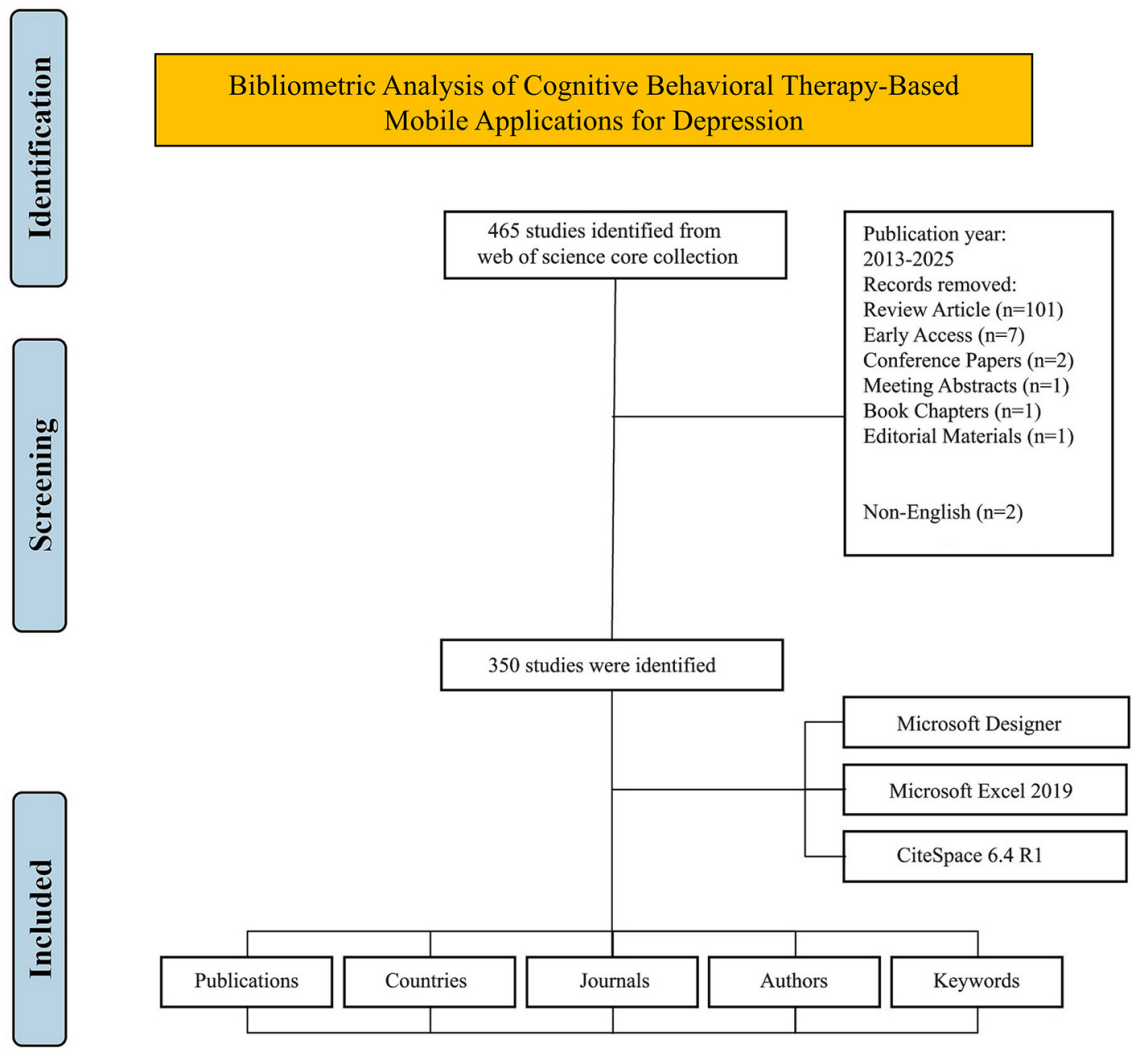
PRISMA 2020 flow diagram of study identification and selection in the WoSCC search.

This analysis of the WoSCC included peer-reviewed original research articles. Reviews, conference papers, meeting abstracts, book chapters, editorial materials, letters to the editor, retracted articles, duplicate publications, and non-English language articles were excluded from this analysis. The rationale for excluding non-English language articles was to maintain the linguistic homogeneity of the corpus, a methodological requirement for ensuring the accuracy of text-based analyses, such as keyword co-occurrence and thematic clustering, performed by the bibliometric software. Figure 1 shows the PRISMA 2020 flow diagram that summarizes identification, screening, eligibility, and inclusion of records retrieved from the WoSCC.

### 2.3 CiteSpace parameters and visualization for WoSCC

We utilized CiteSpace 6.4 R1 to analyze the bibliographic data exported from WoSCC. Data files containing full bibliographic records and cited references were exported as plain text files labeled “download_XXX.txt.” The software was employed to construct visual knowledge maps through several analytical procedures, including temporal slicing, thresholding, and pruning (12, 13). CiteSpace identifies dominant research patterns, emerging topics, and research frontiers by applying core algorithms such as burst detection, betweenness centrality calculation, and heterogeneous network analysis (14). In the resulting visualizations, nodes represent items such as authors, institutions, countries, or keywords. Node size reflects frequency or citation count, while node color indicates the year of publication or citation occurrence. Nodes encircled by a purple ring indicate high betweenness centrality, signifying pivotal points or significant hotspots within the research domain.

## 3 Scientometric results based on WoSCC

### 3.1 Distribution of WoSCC articles by publications and citations

**Figure 2:**
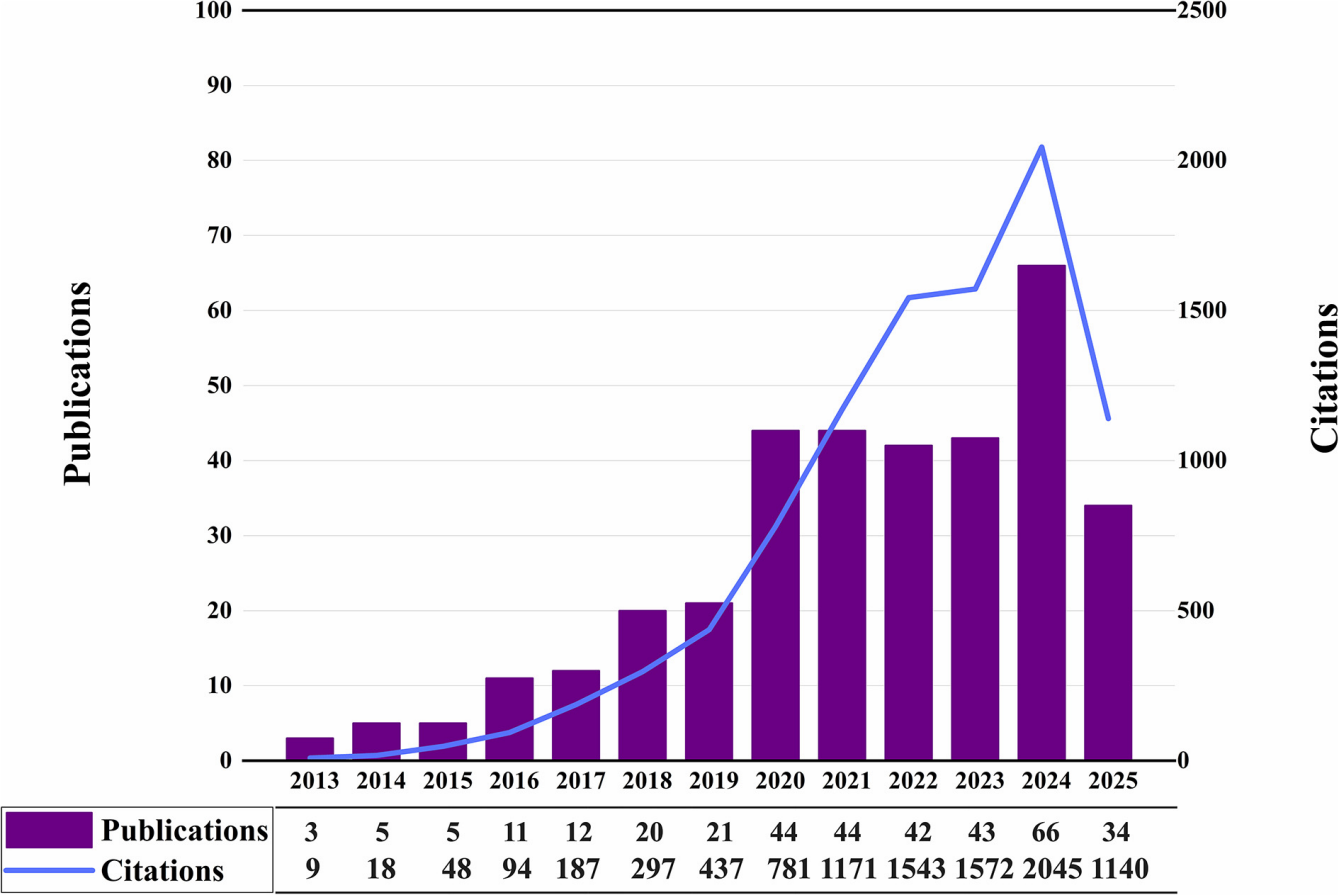
The number of publications and citations each year from 2013 to 2025.

A total of 465 articles were retrieved from WoSCC; after rigorous screening, 350 articles (75.3%) remained for analysis. Among these, the earliest publication dated from 2013, resulting in a 12-year observation period for the WoSCC-based scientometric analysis. Figure 2 illustrates the annual numbers of publications and citations from 2013 to 2025. Annual publications increased gradually from 3 in 2013 to double digits by 2016, followed by a relatively steady climb through 2019. A pronounced surge then appeared between 2020 (44 articles) and 2024 (66 articles), reflecting heightened research interest. The 2025 count currently stands at 34 publications, a figure that represents only data collected up to June.

Citation patterns show a similar upward trajectory: annual citations rose from 9 in 2013 to 2045 in 2024, with especially rapid growth between 2020 (781 citations) and 2024 (2045 citations). This trend underscores the field's increasing scholarly influence.

### 3.2 Research cooperation network in WoSCC

#### 3.2.1 WoSCC co-authorship network

The co-authorship network was visualized using CiteSpace with one-year time slices and the g-index criterion (*k* = 25) (15, 16). The tree-ring history visualization method was applied, where each node represents an author, and the rings indicate the author's cumulative publication record over time. Collaboration between authors is depicted through connecting lines, with the color of each line representing the first year of their collaboration.

**Figure 3:**
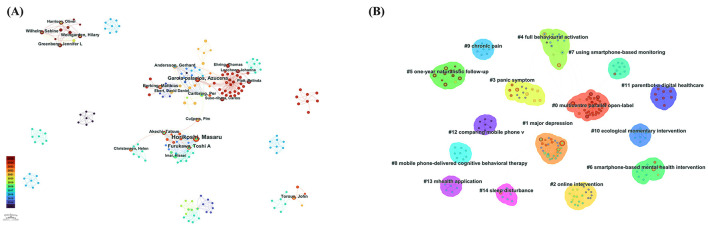
**(A)** The author collaboration network, showing co-authored relationships with connecting lines. **(B)** The clustered co-authorship network based on title keywords, illustrating 15 thematic clusters numbered from #0 to #14, with lower numbers indicating larger clusters.

Figure 3A shows the main connected component of the author collaboration network, which comprises 426 nodes (authors) and 875 collaborative links, with a low network density (0.0097). The most productive authors within this network are Horikoshi Masaru with 11 publications, Garcia-Palacios Azucena with 8 publications, and Furukawa Toshi A with 7 publications. The visualization clearly illustrates distinct research groups with limited inter-group collaborations, indicating that cross-institutional or interdisciplinary cooperation in this field remains relatively uncommon and could benefit from enhancement.

Figure 3B presents the clustered co-authorship network based on title keywords. This clustering analysis was conducted using CiteSpace's log-likelihood ratio method to identify thematic groups (17). The quality of the resulting clusters was assessed using two widely recognized metrics: modularity (*Q*-value) and mean silhouette (*S*-value). Modularity (*Q*) measures the extent to which a network is divisible into clearly defined clusters, with a *Q*-value >0.3 typically indicating significant clustering structure (18). The mean silhouette (*S*) value reflects the consistency or homogeneity of the clusters, with values above 0.7 suggesting high cluster quality (19, 20). In this study, the clustering results yielded a *Q*-value of 0.9224 and an *S*-value of 0.9849, indicating a highly robust and meaningful clustering structure.

The resulting analysis identified 15 thematic clusters numbered from #0 to #14, where lower numbers represent larger clusters. The primary themes include multicenter clinical trials, major depressive disorder, online and smartphone-based psychological interventions, cognitive-behavioral approaches delivered via mobile phones, chronic pain management, and sleep disturbances. These themes underline the current focus of author groups on mobile technology-driven mental health interventions, clinical trials, and digitally facilitated treatment protocols.

#### 3.2.2 WoSCC co-country collaboration network

**Figure 4:**
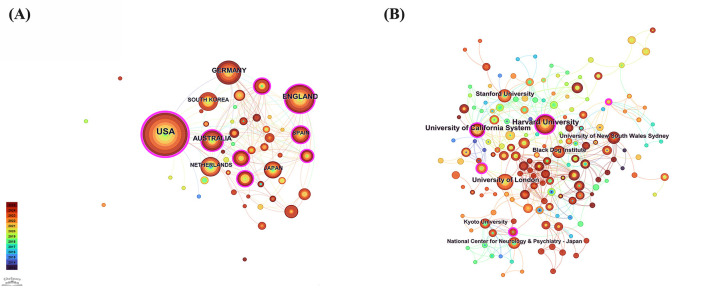
**(A)** The map illustrates the network of country cooperation in this field, where the connecting lines signify the interactions between countries. **(B)** Map of the institute cooperation network in this field, with connecting lines representing institution interactions.

Figure 4A shows the national collaboration network in this field. The network includes 49 countries (nodes) and 161 collaborative links, with a density of 0.1369. The United States (151 publications), England (67 publications), and Germany (52 publications) are the most prolific contributors.

Betweenness centrality—a metric reflecting how often a country lies on the shortest path between other countries—exceeds 0.10 for several key players, underscoring their role as bridges in the international research landscape (21, 22). The United States leads markedly with a centrality of 0.55, highlighting its pivotal position in global partnerships. Switzerland (0.20), England (0.19), and Australia (0.17) also demonstrate high centrality values, indicating their significant roles in connecting disparate research clusters.

#### 3.2.3 WoSCC co-institution collaboration network

Figure 4B presents the institutional cooperation network, which comprises 237 nodes and 623 collaborative links, yielding a network density of 0.0223. The most prolific institutions in terms of publication count were Harvard University (33 publications), the University of California System (26 publications), and the University of London (21 publications). The University of California System (0.16), the Institute of Statistical Mathematics, Japan (0.16), and Harvard University (0.13) exhibited high betweenness centrality, underscoring their pivotal roles in bridging institutional research efforts across the globe.

### 3.3 Co-citation analysis in WoSCC

#### 3.3.1 WoSCC author co-citation network

**Figure 5:**
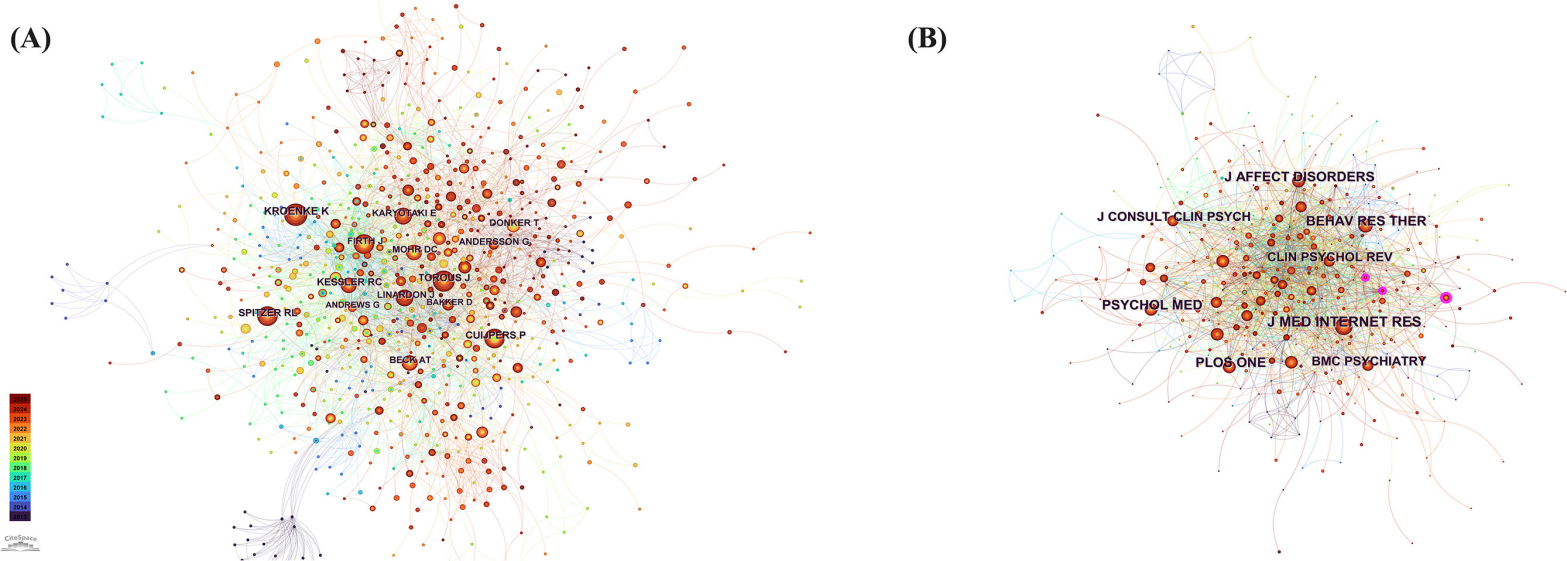
**(A)** Author co-citation network, depicting authors most frequently cited together and the central nodes that link research clusters. **(B)** Journal co-citation network, illustrating the journals most often cited in combination and their roles in structuring the domain's literature.

Figure 5A illustrates the author co-citation network. Kroenke K leads the field with 123 co-citations, followed by Cuijpers P (90), Spitzer RL (80), Torous J (79), and Kessler RC (70). High betweenness centrality nodes—Andersson G (0.17), Beck AT (0.16), Andrews G (0.16), Cohen J (0.12), Kroenke K (0.10), Carlbring P (0.10), and Bangor A (0.10)—serve as critical connectors, linking disparate author clusters and facilitating the flow of key ideas across the network.

#### 3.3.2 WoSCC journal co-citation network

Figure 5B shows the journal co-citation network. The Journal of Medical Internet Research is the most frequently co-cited (280 citations), followed by the Journal of Affective Disorders (214) and Psychological Medicine (184). Acta Psychiatrica Scandinavica (centrality = 0.12), Clinical Psychology & Psychotherapy (0.11), and American Psychologist (0.10) rank highest in betweenness centrality, underscoring their pivotal roles in bridging subfields and shaping the discipline's intellectual structure.

#### 3.3.3 WoSCC reference co-citation clusters and citation bursts

**Figure 6:**
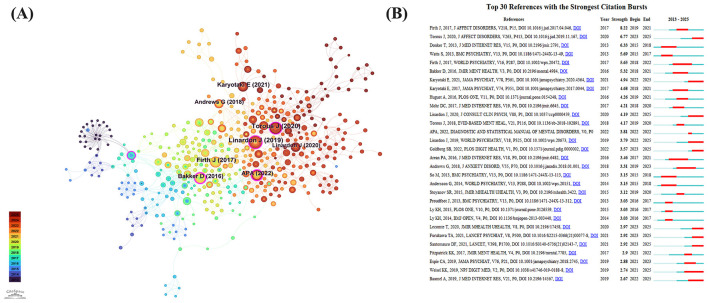
**(A)** Network visualization of reference co-citation analysis, highlighting the interconnectedness of influential studies within this field. **(B)** Top 30 references with strongest citation bursts, reflecting periods of increased research attention.

Figure 6A illustrates the reference co-citation network, visually representing the interrelationships among highly cited references within this field. The most frequently co-cited references included the study by Torous et al. (23), cited 27 times, which systematically reviewed dropout rates in randomized controlled trials of smartphone apps for depressive symptoms, highlighting the significant challenge of maintaining user engagement in app-based mental health interventions. The second most cited study by Linardon et al. (24), cited 25 times, provided a comprehensive meta-analysis demonstrating that app-supported smartphone interventions can effectively reduce depressive and anxiety symptoms, emphasizing the promise of mobile apps in bridging mental healthcare gaps. Firth et al. (25), cited 22 times, conducted the first meta-analysis evaluating the efficacy of smartphone-based mental health interventions for depressive symptoms, concluding that smartphone applications significantly reduced depressive symptoms compared to control conditions. References with the highest betweenness centrality included Bakker et al. (26), with a centrality of 0.28, whose work evaluated mental health apps based on CBT principles and underscored their effectiveness in managing depression and anxiety symptoms through evidence-based interventions and usability testing. The second central reference was the American Psychiatric Association's Diagnostic and Statistical Manual of Mental Disorders, Fifth Edition, Text Revision (DSM-5-TR, 2022; centrality = 0.20) (27), which introduced significant updates in diagnostic criteria and new diagnostic entities, reflecting the latest clinical and research insights in mental disorders, including mood disorders and grief-related conditions. Watts et al. (28), with a centrality of 0.13, conducted an RCT comparing CBT delivered via mobile phones vs. computers, finding both modes significantly improved depressive symptoms and suggesting mobile phone-based CBT as a flexible, accessible alternative for depression treatment.

Figure 6B presents the top 30 references with the strongest citation bursts between 2013 and 2025, indicating periods of heightened research attention and shifting focus within this domain.

**Figure 7:**
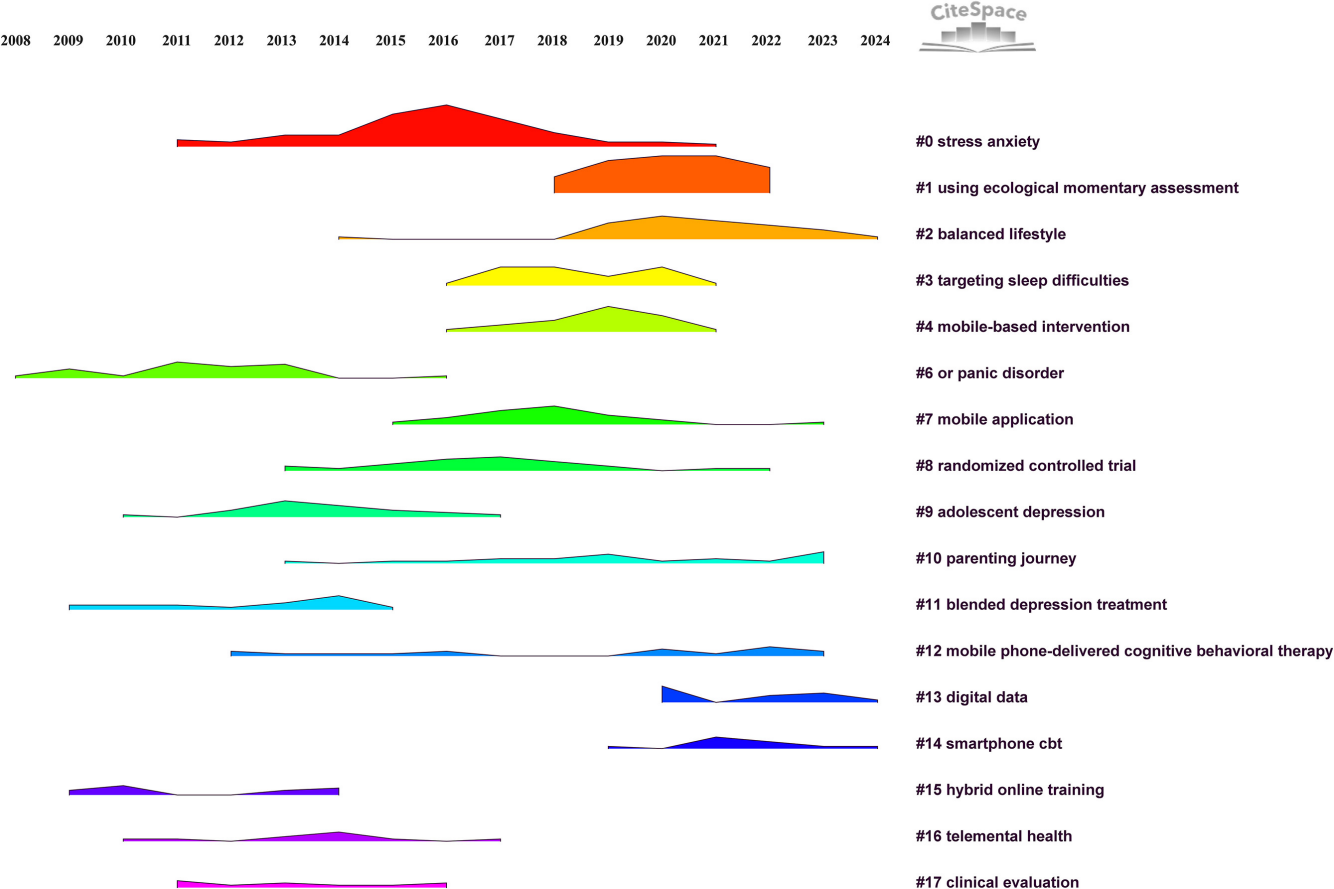
Clustering landscape of reference co-citation network based on title keywords, illustrating the evolution of research hotspots and thematic trends from 2013 to 2025.

Reference co-citation clustering demonstrated strong modularity (*Q* = 0.7975) and high silhouette scores (*S* = 0.8932), indicating clearly delineated and meaningful clusters. Specifically, a *Q*-value near 0.8 indicates robust clustering modularity, reflecting strong internal cohesion and clear boundaries among clusters, while an *S*-value above 0.7 confirms high homogeneity within each cluster. Figure 7 presents the clustering landscape, revealing 18 distinct clusters (#0–#17, with lower numbers representing larger clusters). These clusters primarily covered themes such as stress and anxiety management, ecological momentary assessment methods, sleep intervention strategies, mobile-based interventions, randomized controlled trials, adolescent depression, and mobile-delivered CBT, highlighting a dynamic evolution of research hotspots from 2013 to 2025.

### 3.4 Keyword analysis in WoSCC

#### 3.4.1 WoSCC keyword co-occurrence and clusters

Keyword co-occurrence analysis was conducted using annual time slices (*k* = 25), identifying 353 nodes and 1,289 links, with a network density of 0.0207. Table 2 presents the top ten keywords ranked by frequency alongside their occurrence counts, and the top ten keywords ranked by betweenness centrality alongside their centrality scores. The log-likelihood ratio clustering algorithm revealed 13 thematic clusters that capture focal areas such as mobile applications, CBT, smartphone-based interventions, digital health strategies, and the integration of artificial intelligence into mental health interventions.

**Table 2:** Top ten keywords by frequency and betweenness centrality.

**Rank**	**Frequency**	**Keywords**	**Centrality**	**Keywords**
1	183	Cognitive behavioral therapy	0.14	Disorders
2	135	Depression	0.13	Psychotherapy
3	112	Meta-analysis	0.13	Adolescents
4	82	Anxiety	0.10	Depression
5	78	Mental health	0.10	Anxiety
6	61	Efficacy	0.10	Care
7	55	Symptoms	0.10	Anxiety disorders
8	48	Randomized controlled trial	0.10	Internet
9	48	Validation	0.09	Health
10	44	Validity	0.09	Intervention

**Figure 8:**
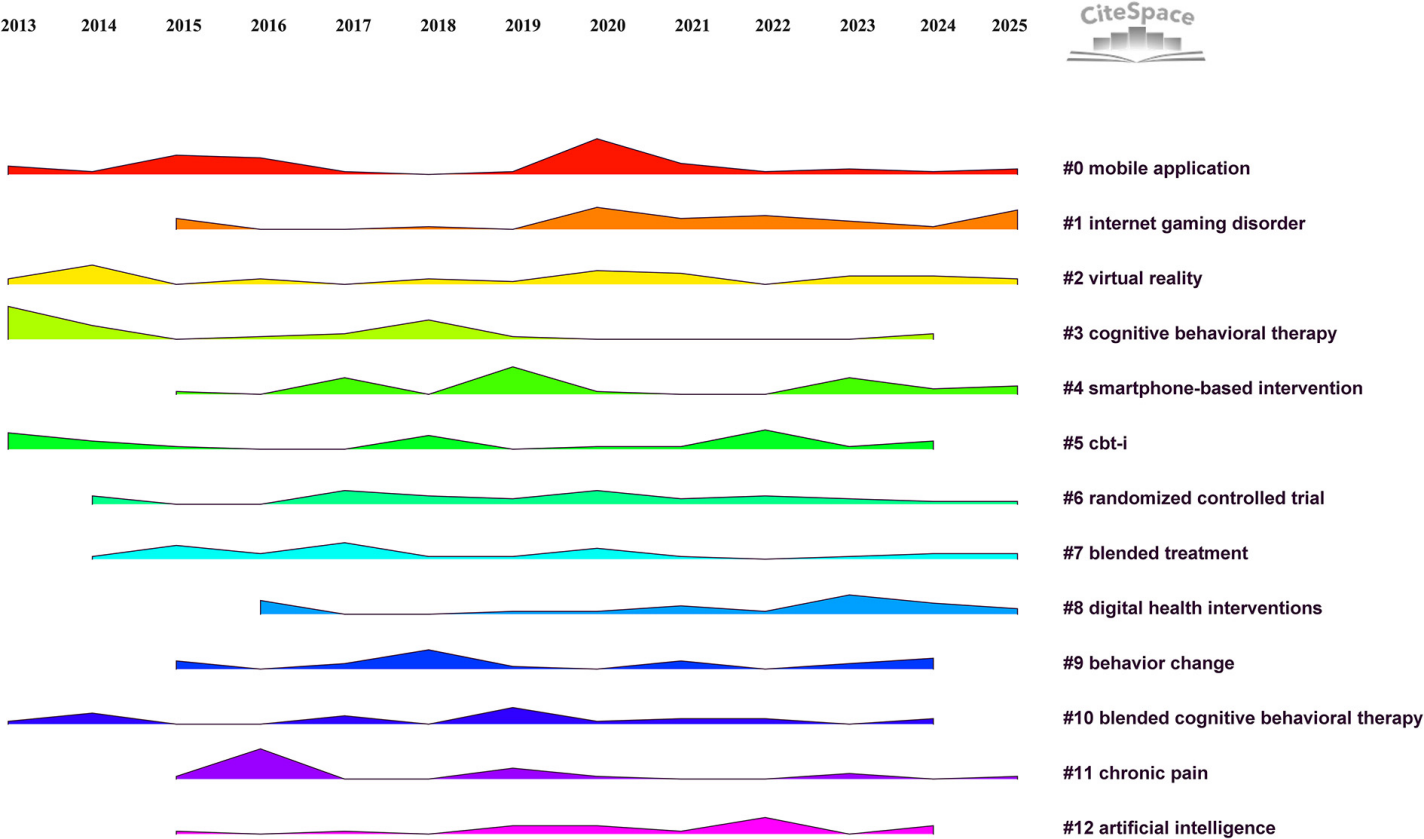
Thematic keyword clusters illustrating the research evolution from 2013–2025, where lower numbers denote larger clusters.

Figure 8 shows the landscape of keyword co-occurrence clusters, illustrating the evolving focus of research from 2013 to 2025. The visualized landscape reveals how research priorities have shifted over time, with a continued emphasis on mobile mental health interventions and a growing interest in new technologies like artificial intelligence.

#### 3.4.2 WoSCC keywords with citation bursts

**Figure 9:**
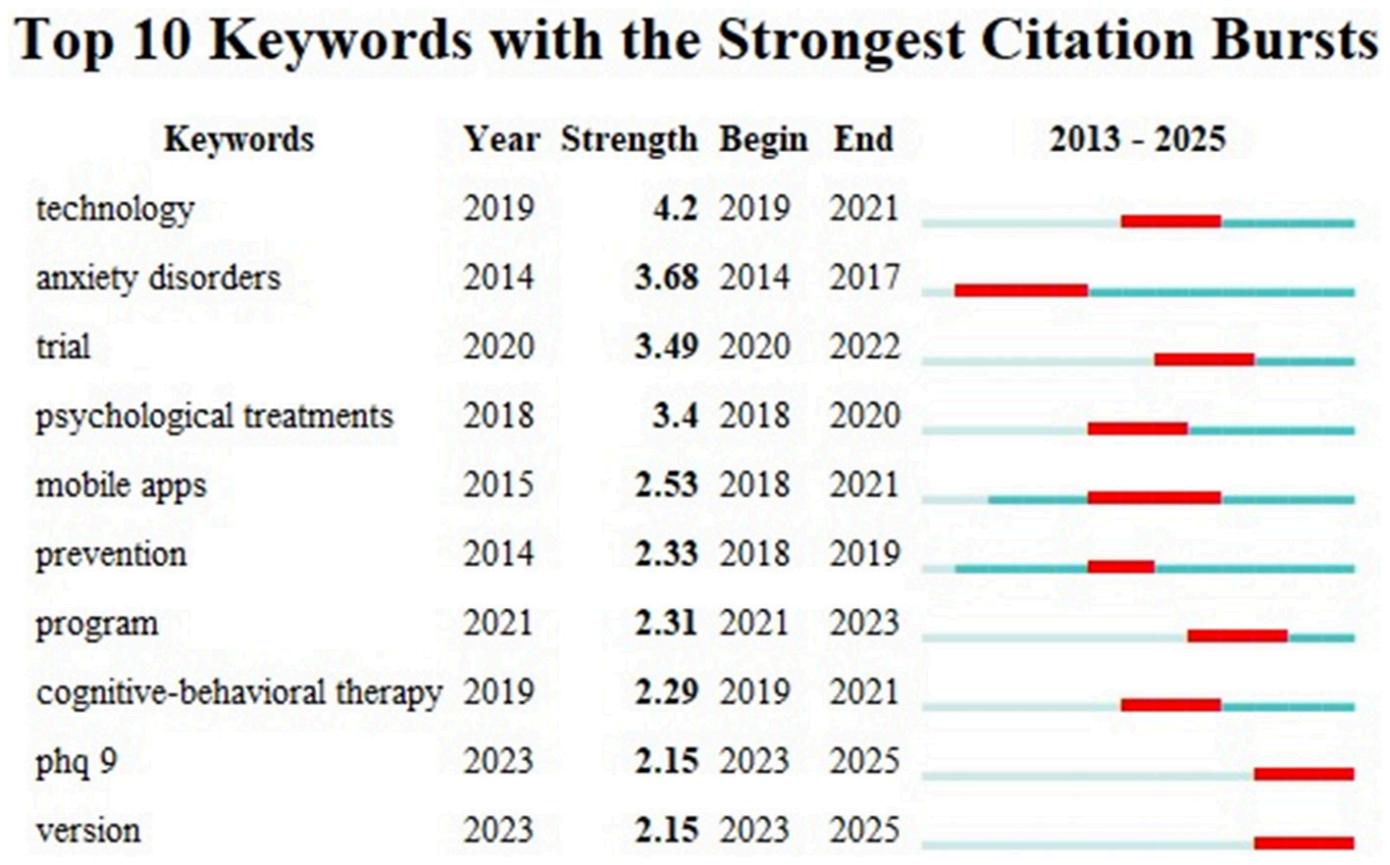
The top 10 burst keywords from 2013 to 2025.

Keywords experiencing citation bursts indicate significant emerging topics within a specific field. Figure 9 analyzes the top 10 keywords with the strongest citation bursts from 2013 to 2025, reflecting significant shifts and trends in research. The keyword “technology,” with a burst strength of 4.2 from 2019 to 2021, highlights the growing interest in integrating technology with CBT for depression treatment. Similarly, “anxiety disorders” (burst strength 3.68, 2014–2017) and “mobile apps” (2.53, 2018–2021) underscore the increasing research focus on digital interventions addressing anxiety and leveraging mobile platforms for mental health. Other prominent bursts, such as “trial” (3.49, 2020–2022) and “psychological treatments” (3.4, 2018–2020), point to the growing empirical focus on evaluating the effectiveness of digital mental health treatments. These bursts reflect the evolving research priorities in digital mental health interventions. Other keywords experiencing citation bursts include “prevention” (burst strength 2.33, 2018–2019), “program” (2.31, 2021–2023), “cognitive-behavioral therapy” (2.29, 2019–2021), “phq 9” (2.15, 2023–2025), and “version” (2.15, 2023–2025). The rise of “prevention” reflects an increasing emphasis on proactive mental health care and early intervention strategies. “Program” points to the growing focus on structured, evidence-based intervention programs for managing depression. “Cognitive-behavioral therapy,” a cornerstone of many mobile applications, continues to gain importance in digital mental health treatments. The recent surge in “phq 9,” a well-known tool for assessing depression, reflects growing efforts to measure and track the effectiveness of digital mental health solutions. Finally, “version” refers to the evolving nature of mobile applications, particularly the updates and improvements being made to enhance their therapeutic effectiveness.

## 4 Clinical progress trends based on pubmed

### 4.1 Methods and search strategy

PubMed is a widely recognized biomedical literature database managed by the U.S. National Library of Medicine (NLM) (29). It specializes in biomedicine and life sciences literature, offering extensive coverage and high-quality indexing of clinical studies, making it particularly suitable for analyzing clinical trends and advancements. Its robust search functionalities, including MeSH terms and advanced search fields, facilitate precise retrieval of relevant clinical trial articles. Understanding clinical progress trends through clinical trials is crucial, as these studies provide essential evidence for the safety, efficacy, and clinical applicability of interventions. Clinical trials play a pivotal role in translating theoretical research findings into practical therapeutic applications, thereby enhancing patient outcomes and guiding evidence-based clinical practice (30).

**Figure 10:**
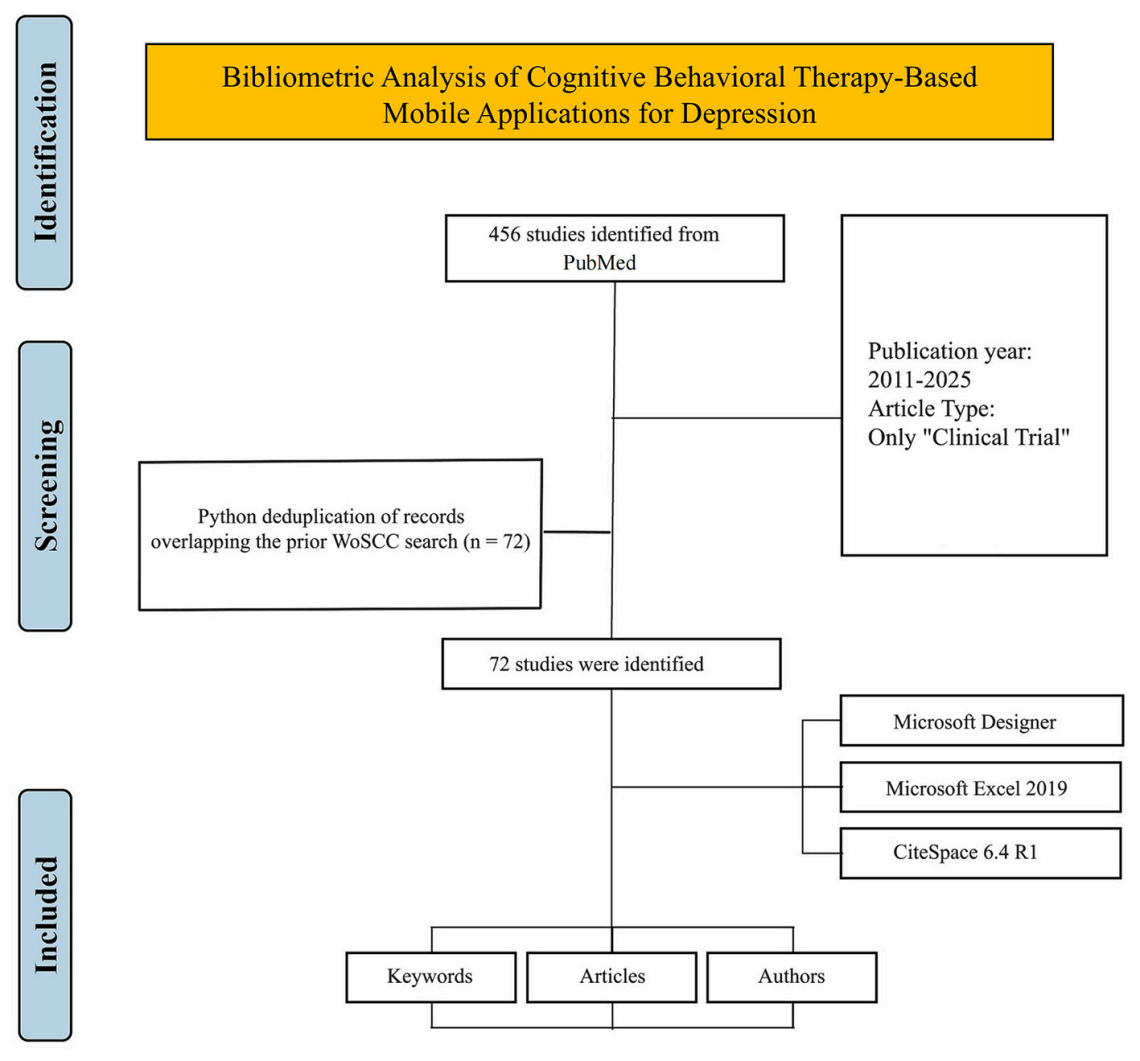
PRISMA 2020 flow diagram of study identification and selection in the PubMed clinical-trial subset.

Following the comprehensive analysis of data from the WoSCC, an analysis of clinical trials was conducted to further explore clinical trends in cognitive behavioral therapy-based mobile applications for treating depression. We developed a tailored search strategy according to PubMed's specific retrieval rules. The complete, executable search query was: ((“Cell Phone”[Mesh] OR smartphone^*^[tiab] OR “smartphone application”[tiab] OR “smartphone applications”[tiab] OR “mobile application”[tiab] OR “mobile applications”[tiab] OR “mobile app”[tiab] OR “mobile apps”[tiab] OR “mobile phone app”[tiab] OR “mobile phone apps”[tiab] OR “mental health app”[tiab] OR “mental health apps”[tiab]) AND (“Depression”[Mesh] OR “Depressive Disorder, Major”[Mesh] OR depress^*^[tiab] OR “major depressive disorder”[tiab] OR MDD[tiab]) AND (“Cognitive Behavioral Therapy”[Mesh] OR “Cognitive Behavioral Therapy”[tiab] OR CBT[tiab] OR chatbot^*^[tiab] OR “conversational agent”[tiab] OR “conversational agents”[tiab])). This search strategy was organized into three primary conceptual components: the first component encompassed terms related to smart devices and mobile applications; the second component covered the target disease, depression; and the third component included terms for cognitive behavioral therapy. The final search query combined these three components using the ‘AND' operator to ensure comprehensive coverage of the target literature. Table 3 presents the detailed PubMed search strategy utilized for this analysis. This strategy was executed from database inception up to June 25, 2025, initially yielding 456 articles. By limiting the results to articles categorized explicitly as “Clinical Trial,” the search was narrowed to 144 relevant publications. To avoid duplication, a comparison between these 144 articles and the previously retrieved 350 articles from WoSCC was performed using Python scripting, resulting in the removal of 72 duplicates. Consequently, 72 unique articles were included in the final analysis. Figure 10 shows the corresponding PRISMA 2020 flow diagram for the PubMed clinical-trial subset used to validate the bibliometric findings. This process underscores the methodological rationale for the multi-database strategy by highlighting the distinct, complementary roles of each database. While the WoSCC is the established standard for scientometric analysis and essential for mapping macro-level knowledge structures based on citation networks, its primary focus is not exhaustive for all specialized clinical intervention literature. Conversely, PubMed serves as the authoritative repository for biomedical and life sciences literature, offering superior, specialized indexing for clinical studies through mechanisms like MeSH terms and the “Clinical Trial” publication type filter. The inherent discrepancy in their indexing focus is precisely what necessitates this complementary approach. The identification of 72 unique clinical trial articles from PubMed—which were not captured in the 350-article WoSCC dataset —provides concrete evidence of this gap. Therefore, the parallel analysis of the PubMed subset facilitates a focused, micro-level examination of clinical translation trends that the WoSCC-based macro-analysis alone would incompletely represent, thereby providing a more comprehensive map of the field from foundational research to practical application.

**Table 3:** Retrieval strategy for pubmed.

**Set**	**Search query**
#1	“Cell Phone”[Mesh] OR smartphone^*^[tiab] OR “smartphone application”[tiab] OR “smartphone applications”[tiab] OR “mobile application”[tiab] OR “mobile applications”[tiab] OR “mobile app”[tiab] OR “mobile apps”[tiab] OR “mobile phone app”[tiab] OR “mobile phone apps”[tiab] OR “mental health app”[tiab] OR “mental health apps”[tiab]
#2	“Depression”[Mesh] OR “Depressive Disorder, Major”[Mesh] OR depress^*^[tiab] OR “major depressive disorder”[tiab] OR MDD[tiab]
#3	“Cognitive Behavioral Therapy”[Mesh] OR “Cognitive Behavioral Therapy”[tiab] OR CBT[tiab] OR chatbot^*^[tiab] OR “conversational agent”[tiab] OR “conversational agents”[tiab]
#4	#1 AND #2 AND #3

### 4.2 Co-authorship

**Figure 11:**
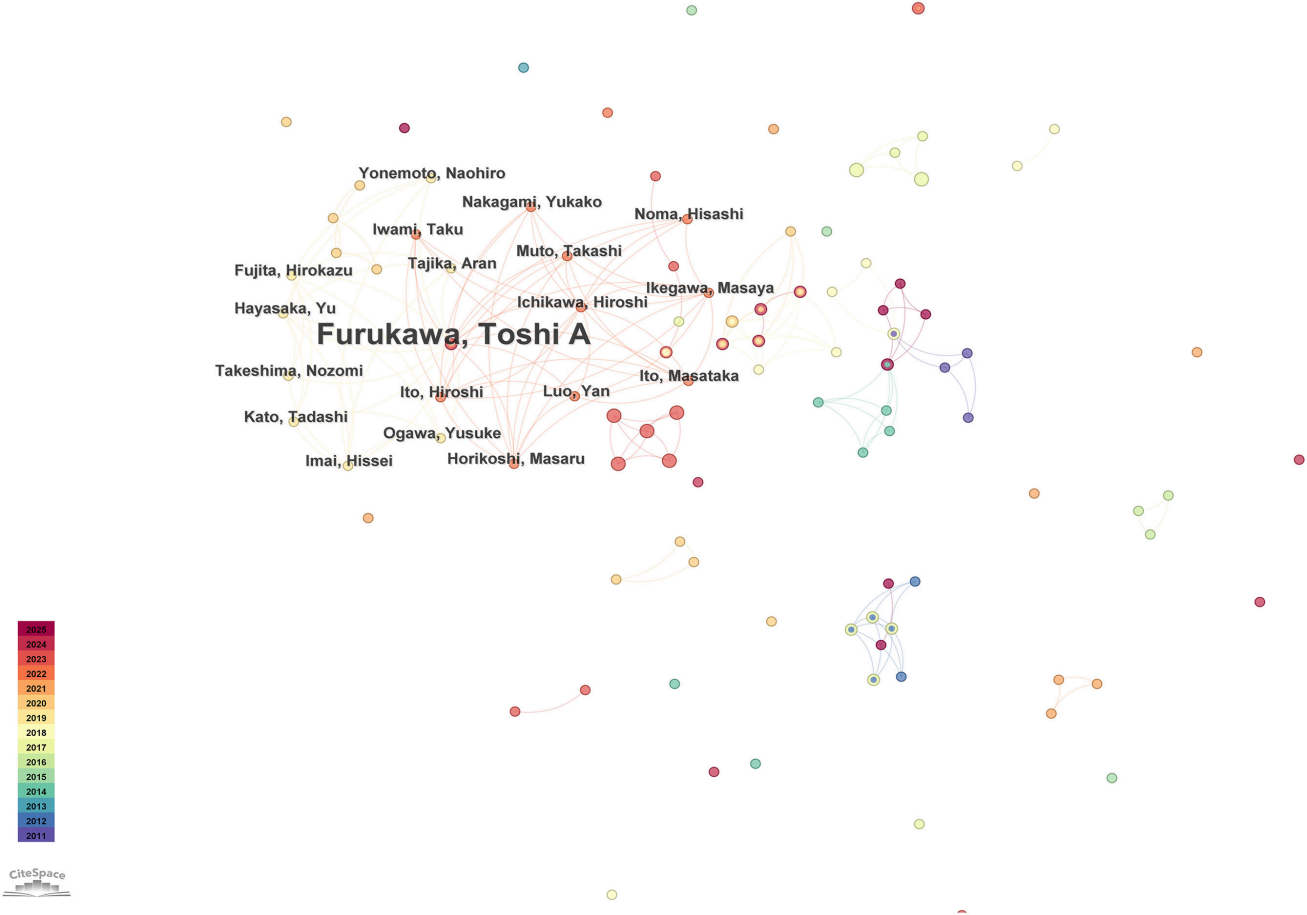
Author co-authorship network based on PubMed clinical trials.

Figure 11 visualizes the main connected component of the author collaboration network based on PubMed data. The most prolific authors were Furukawa, Toshi A, Greeson, Jeffrey M, Gremore, Tina, Levin, Michael E, and Cox, Christopher E, each contributing three publications. Notably, the visualization highlights a noticeable paucity of interdisciplinary and inter-institutional collaborations in clinical trials within this field. Strengthening cooperation across different institutions and disciplines could significantly enrich the scope and robustness of clinical studies. Enhanced collaborative efforts can facilitate resource sharing, improve methodological rigor, broaden participant recruitment, and ultimately produce more generalizable and impactful clinical outcomes.

### 4.3 Keyword analysis

**Figure 12:**
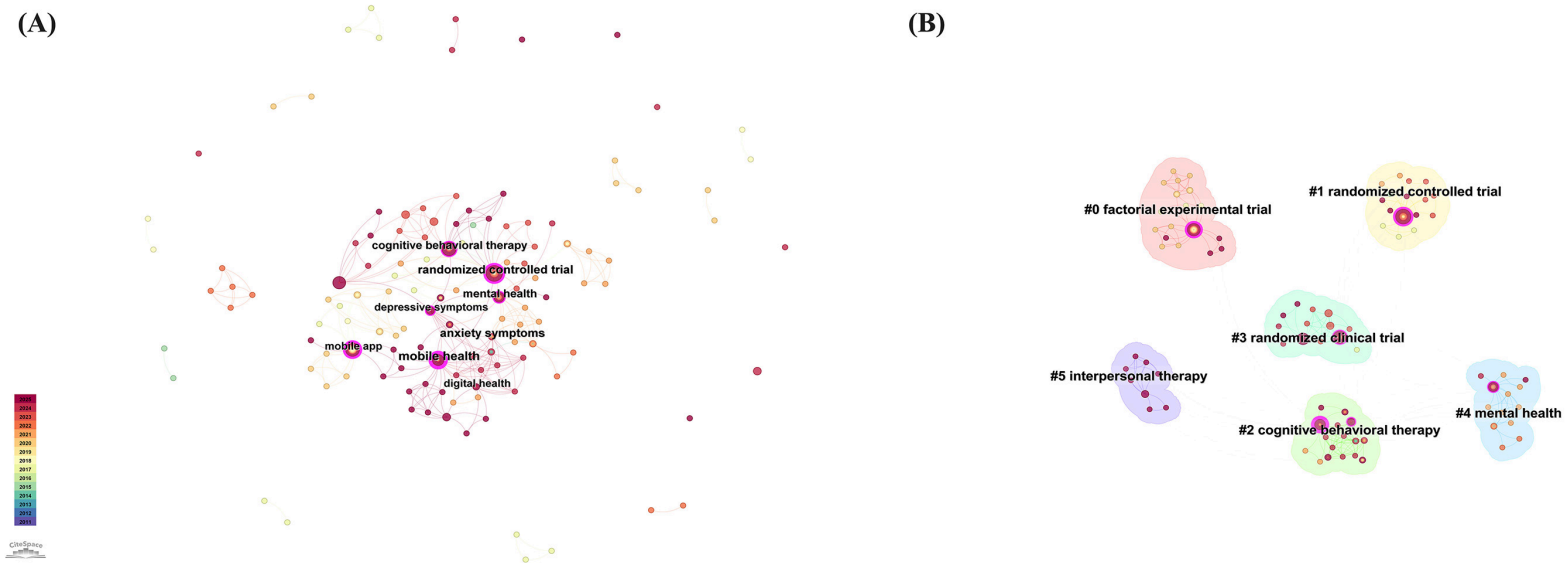
**(A)** Keyword co-occurrence network based on PubMed clinical trials. **(B)** The clustered keyword network based on PubMed clinical trials, illustrating six thematic clusters numbered from #0 to #5, with lower numbers indicating larger clusters.

Figure 12A presents the keyword co-occurrence network based on PubMed data. The most frequently occurring keywords were randomized controlled trial (11 occurrences), cognitive behavioral therapy (8 occurrences), mobile phone (7 occurrences), mental health (7 occurrences), and mobile app (6 occurrences). Keywords exhibiting high betweenness centrality included mobile health (0.45), randomized controlled trial (0.31), and mobile app (0.24). The prominence of these keywords underscores the critical role that randomized controlled trials play in assessing the efficacy of CBT-based mobile applications. RCT are recognized as the gold standard in clinical research due to their ability to minimize bias and produce high-quality evidence, thereby significantly contributing to the development and validation of clinical practices in digital mental health (31).

Figure 12B illustrates keyword clusters derived from the analysis, including randomized controlled trial, factorial experimental trial, cognitive behavioral therapy, mental health, randomized clinical trial, and interpersonal therapy. These clusters represent key research trends and areas of clinical focus within this field. Specifically, the dominance of “randomized controlled trial” and related terms highlights ongoing efforts to rigorously evaluate digital therapeutic interventions. Additionally, clusters such as “interpersonal therapy” indicate expanding clinical interests beyond traditional CBT, suggesting an emerging trend toward integrating multiple therapeutic modalities into mobile mental health applications. Collectively, these keyword clusters reflect a growing research emphasis on methodological rigor, diverse therapeutic approaches, and the clinical validation of mobile interventions for depression treatment.

## 5 Behavioral and psychological research trends based on PsycINFO

### 5.1 Methods and search strategy

PsycINFO is the premier abstracting and indexing database produced by the American Psychological Association and is widely regarded as the most comprehensive resource for peer-reviewed literature in behavioral science and mental health (32). To complement the macro-level analysis based on WoSCC and the clinically oriented search conducted in PubMed, PsycINFO was interrogated through the APA PsycNet platform to capture research on cognitive behavioral therapy–based mobile applications for depression originating from core psychology and behavioral science journals.

Guided by the APA Thesaurus of Psychological Index Terms, we first operationalized the three central constructs of this review—mobile delivery modality, depressive disorders, and cognitive behavioral therapy (33). We then applied a keyword-based strategy that treated the descriptors “mobile phones”, “depression”, and “cognitive behavioral therapy” as terms in the Any Field option, enabling PsycINFO to retrieve records in which these concepts appeared in titles, abstracts, author keywords, or indexing fields and thereby maximizing coverage while minimizing the risk of omitting relevant literature. The search encompassed all records indexed up to June 25, 2025. Inclusion criteria were limited to publicly published literature available in English. All records retrieved by this strategy that met the inclusion criteria were exported and imported into CiteSpace for co-authorship and keyword analyses. The final PsycINFO dataset comprised 20 articles, which served as the basis for the supplementary bibliometric analysis of behavioral and psychological research trends in this section. By incorporating PsycINFO, which indexes a wide range of specialized psychology and behavioral science journals that are not comprehensively represented in WoSCC or PubMed, this study strengthens its methodological rigor and provides a more nuanced view of how CBT-based mobile interventions for depression have been examined within core behavioral and mental health disciplines, thereby enriching the interpretation of the global patterns identified in the WoSCC and PubMed datasets.

### 5.2 Co-authorship

**Figure 13:**
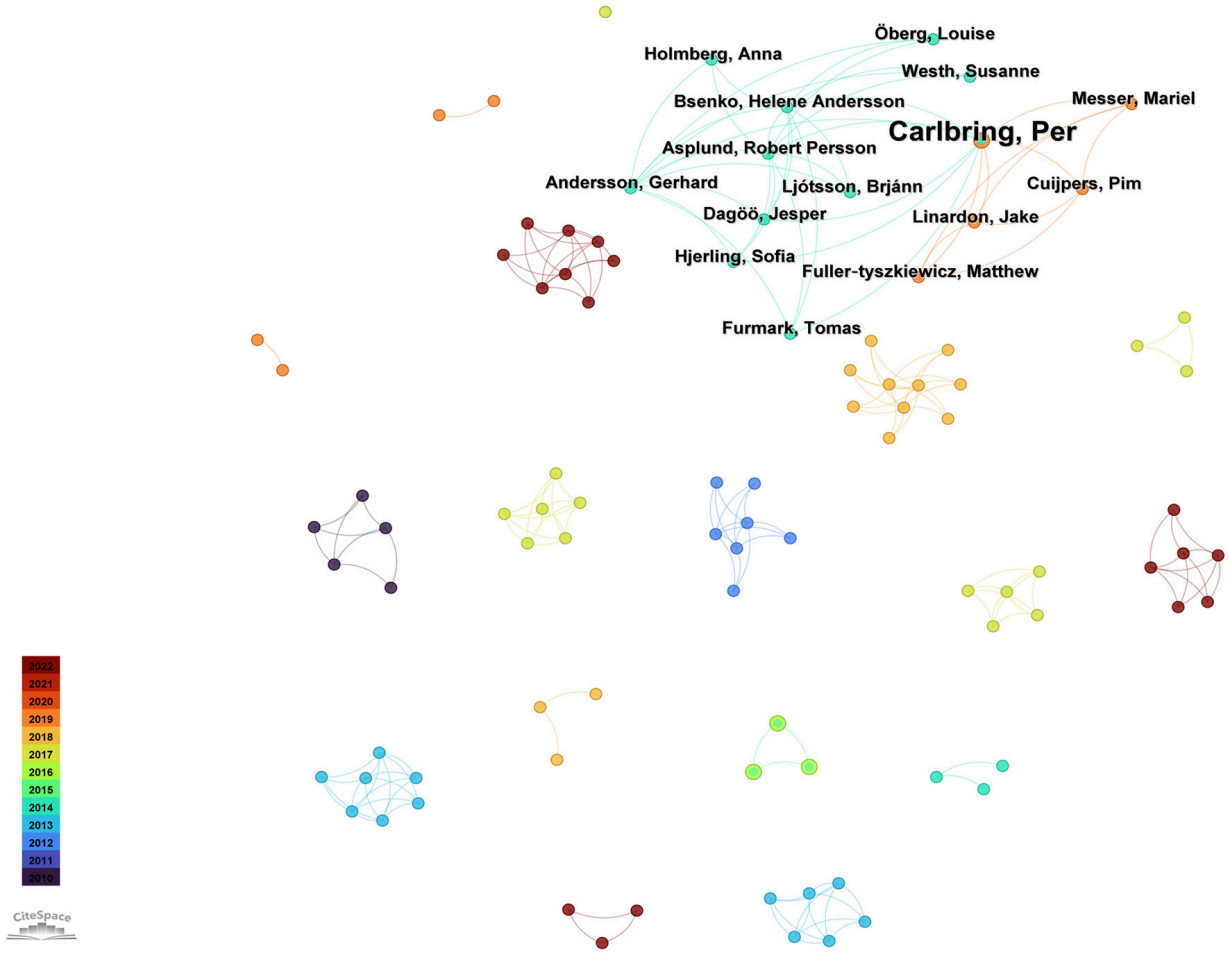
Co-authorship network of CBT-based mobile application studies for depression in the PsycINFO database.

Figure 13 shows the co-authorship network of CBT-based mobile application studies for depression retrieved from the PsycINFO database. Within this relatively small but conceptually focused corpus, Kennard Betsy, Carlbring Per, Kobak Kenneth A, and Mundt James C each contributed two publications and thus emerge as the most prolific authors. The visualization highlights a prominent cluster centered on Carlbring Per that links multiple collaborators across different years, suggesting a sustained and evolving research program in which a stable core team has repeatedly examined digital CBT interventions for depression from a behavioral science perspective. Surrounding this core are several smaller, tightly knit clusters, many of which are represented by warmer colors on the timeline scale and therefore indicate research groups that have entered the field more recently. Rather than a single, densely interconnected network, the PsycINFO map portrays a structure characterized by a leading behavioral research hub accompanied by emerging teams in diverse settings, implying that CBT-based mobile applications for depression are increasingly becoming an established topic within psychology and behavioral health while still leaving room for broader cross-group collaboration.

### 5.3 Keyword analysis

**Figure 14:**
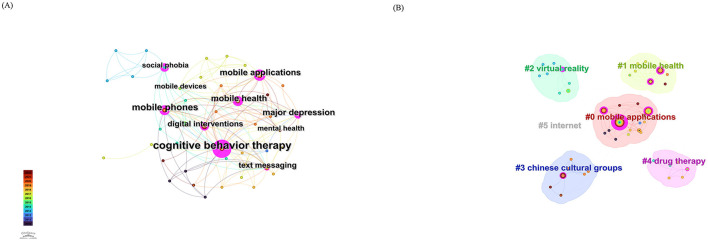
**(A)** Keyword co-occurrence network based on CBT-based mobile application studies for depression in the PsycINFO database. **(B)** The clustered keyword network based on these studies, illustrating the corresponding thematic clusters.

Figure 14A presents the keyword co-occurrence network for CBT-based mobile application studies on depression in the PsycINFO dataset. The most frequent keywords are mobile phones with 18 occurrences, cognitive behavior therapy with 14 occurrences, major depression with 10 occurrences, digital intervention with 4 occurrences, and mobile applications with 4 occurrences. Keywords with the highest betweenness centrality include cognitive behavior therapy with a value of 0.88, mobile phones with 0.32, social phobia with 0.32, major depression with 0.27, and mobile applications with 0.25. Taken together, these high-frequency and high-centrality terms confirm that the PsycINFO corpus focuses on mobile phone-delivered CBT interventions targeting major depressive disorder, while at the same time highlighting the conceptual framing of these tools as digital interventions rather than merely device-based solutions. The emergence of digital intervention as a relatively new but strategically positioned keyword underscores the growing recognition of CBT-based mobile tools as a distinct branch within the broader field of digital mental health. The prominence of social phobia as a bridging term further suggests that mobile CBT interventions developed for depression are often embedded within transdiagnostic or comorbidity-focused programs, which reflects the clinical reality that depressive and anxiety symptoms frequently co-occur and can be addressed through shared cognitive behavioral mechanisms.

Figure 14B visualizes the clustering structure of PsycINFO keywords, which can be broadly summarized by six thematic clusters labeled mobile applications, virtual reality, mobile health, Chinese cultural groups, drug therapy, and Internet. The mobile applications and mobile health clusters indicate that the PsycINFO literature anchors CBT-based interventions within the larger mHealth ecosystem, where smartphone apps and mobile services function as core delivery platforms for psychological treatment. The recurrence of virtual reality as a distinct cluster, consistent with the WoSCC keyword analysis, points to a convergent research trend in which immersive and interactive technologies are increasingly integrated with CBT-based mobile interventions in order to enhance exposure, engagement, and skill acquisition. The Chinese cultural groups cluster is particularly noteworthy, as it suggests a growing body of work that adapts and evaluates digital CBT tools among Chinese populations and emphasizes the importance of cultural context, language, and health system characteristics in shaping the uptake and effectiveness of mobile interventions. The presence of drug therapy and Internet clusters indicates that CBT-based mobile applications are often studied alongside pharmacological treatment and web-based platforms, reinforcing the view that digital CBT functions as part of a multimodal care framework that spans face-to-face, online, and mobile modalities.

## 6 Discussion

The primary findings of this bibliometric analysis revealed several important insights into global research trends concerning CBT-based mobile applications for depression. The annual number of publications demonstrated a steadily increasing trend, reaching a peak of 66 articles in 2024. Despite this growth, the number of publications remained below 100 per year, signifying that this research area is still emerging and possesses considerable potential for future expansion. The first significant publication by Watts et al. (28), published in February 2013 in BMC Psychiatry, laid foundational work through a randomized controlled trial comparing traditional computer-based CBT with mobile application-based CBT. This pioneering study demonstrated the feasibility and effectiveness of mobile applications in delivering CBT for depression, highlighting their potential advantages in accessibility and convenience over traditional formats. Opportunities exist to further advance this field by integrating enhanced modalities such as MBCT and ACT into mobile applications, thereby enhancing treatment accessibility and effectiveness (34, 35). Strengthening international and interdisciplinary collaborations could also significantly boost research output and innovation.

In examining national and institutional contributions, the United States emerged as the leading country in terms of both publication volume and centrality, underscoring its role as a pivotal player in the global research landscape. The high centrality suggests that the United States not only contributes substantially but also effectively facilitates international collaboration, likely driven by its robust research infrastructure and established academic networks. European countries, notably England, Germany, and Switzerland, also prominently featured, indicating strong European involvement and cooperation. Australia was also identified as a nation with significant contributions and high betweenness centrality, underscoring its vital role in facilitating cooperation among various countries. However, the visualized cooperation network highlighted a significant gap in collaboration involving Asian nations, as well as Southern Hemisphere countries excluding Australia, suggesting an urgent need for fostering international collaborations with these regions to diversify research perspectives and enhance global research integration.

Institutionally, Harvard University, the University of California System, and the University of London were the most prolific contributors. The prominence of these institutions, along with Stanford University, reinforces the dominance of American and European research organizations in this domain. This reflects limited representation from developing countries, emphasizing the importance of inclusive collaborations between leading and emerging research institutions globally. Promoting partnerships between core research institutions and peripheral entities could facilitate a more inclusive and influential global research community.

Author collaboration analysis identified three influential researchers: Horikoshi Masaru, Garcia-Palacios Azucena, and Furukawa Toshi A. Visual analysis of the co-authorship network illustrated extensive cooperation between Horikoshi Masaru and Furukawa Toshi A, as well as substantial connectivity involving Garcia-Palacios Azucena. Despite these notable collaborations, the visualized network reveals that overall author collaboration remained largely fragmented and limited, with many isolated research clusters. This fragmentation underscores the necessity of enhancing collaboration among authors, institutions, and countries. Single-disciplinary approaches are insufficient for addressing the complex challenges associated with mood disorders, and robust multidisciplinary cooperation is essential to advance understanding and intervention strategies in this critical research area.

The keyword clustering analysis identified several key themes that represent the focal areas and interdisciplinary trends within CBT-based mobile application research. The largest cluster, “mobile application,” highlights the critical role of smartphone-based interventions in broadening access to CBT. Studies within this cluster consistently demonstrate that mobile health applications grounded in CBT principles effectively manage depression symptoms, offering advantages such as cost-effectiveness (36), anonymity (37), and user flexibility (38). Empirical evidence supports their therapeutic value, showing significant reductions in depression and anxiety compared to traditional (39). The prominence of this cluster illustrates a shift toward integrating psychological expertise with technological innovation to optimize mobile mental health services. The cluster “internet gaming disorder” reveals an emerging interdisciplinary focus, extending CBT applications beyond mood disorders to address behavioral addictions linked to digital technologies. Researchers have adapted CBT principles within mobile platforms to treat problematic internet gaming, recognizing its associations with anxiety, depression, and maladaptive behaviors. Studies have demonstrated the effectiveness of CBT-based interventions for internet gaming disorder (40), highlighting the efficacy of combining CBT with mobile applications. This suggests that CBT is not only effective for managing anxiety and depression but can also offer solutions for other complex clinical issues. Exploring the integration of CBT with digital technologies for a broader range of emotional and behavioral disorders appears to be a significant emerging research trend. This trend signifies an expansion of digital CBT research into new domains, blending expertise from addiction medicine, adolescent psychology, and digital health. Similarly, the cluster “virtual reality” underscores innovative efforts to enhance CBT through immersive technologies. Virtual reality (VR) allows for realistic exposure scenarios and interactive environments, significantly enhancing user engagement and therapy efficacy (41). Studies indicate VR-assisted CBT interventions can achieve comparable or superior outcomes to standard therapy, particularly in anxiety-related conditions (42). This cluster's formation highlights increasing collaboration among mental health professionals, engineers, and human-computer interaction experts, marking a promising convergence of CBT with advanced digital therapeutics.

The evolution of keyword bursts further reflects the shifting research priorities in digital CBT interventions. Initially, early research primarily focused on disorders themselves, notably “anxiety disorders,” reflecting the initial intent to validate digital interventions for established clinical conditions. Gradually, the research emphasis shifted from specific disorders to exploring and validating digital therapeutic modalities themselves, illustrated by keyword bursts such as “technology,” “mobile apps,” and “trial.” This transition demonstrates researchers' growing interest in systematically evaluating the feasibility, efficacy, and practical integration of digital interventions into mental healthcare. More recently, keywords such as “prevention,” “program,” and “version” have emerged prominently, indicating an emphasis on structured intervention approaches, preventive mental healthcare strategies, and iterative refinement of digital applications based on user feedback. The frequent occurrence of “PHQ-9” further exemplifies rigorous outcome assessment becoming a standard expectation, enhancing confidence in the clinical efficacy and acceptability of CBT-based mobile interventions. Collectively, these keyword trends depict an ongoing maturation of research, transitioning from initial validation of digital interventions toward optimization and systematic integration into established healthcare practices. This trajectory emphasizes continuous technological innovation, rigorous empirical evaluation, and responsiveness to user-centered design principles. The interdisciplinary nature and increasing empirical support for digital CBT applications position them prominently within future mental healthcare frameworks.

Citation-burst analysis reveals the moments when certain studies suddenly attracted intense attention, thereby pinpointing emerging hotspots and paradigm-shifting contributions within the broader field of CBT-based mobile mental health interventions. Among the thirty references exhibiting the strongest bursts, most address the development, efficacy, and engagement challenges of smartphone-delivered CBT for depression and anxiety, collectively mapping the trajectory from early feasibility studies to large-scale meta-analyses and user-experience evaluations. Firth et al. (43) demonstrate that smartphone interventions yield significant reductions in depressive symptoms compared to control conditions. Torous et al. (23) show that attrition in depression-app trials can approach half of all participants, underscoring the need to bolster user engagement. Donker et al. (44) reveal that only a handful of commercially available mental health apps adhere to evidence-based principles, calling for more rigorous validation. Watts et al. (28) report that CBT delivered via mobile app produces clinical improvements on par with computer-based programs, affirming its flexibility and accessibility. A second analysis by Firth et al. (25) confirms moderate effect sizes for app-based depressive symptom reduction and cautions against untested commercial offerings. Bakker et al. (26) outline concrete, evidence-based design recommendations for future mental health apps, emphasizing the integration of core CBT techniques. Karyotaki et al. (45) establish that Internet-delivered CBT produces robust symptom relief in large samples, illustrating scalability. An earlier review by Karyotaki et al. (46) similarly affirms the efficacy of unguided online CBT for depression. Huguet et al. (47) find that most depression apps lack fundamental CBT or behavioral activation components and suffer from poor usability and privacy safeguards. Mohr et al. (48) illustrate that a suite of focused, skills-based apps can achieve both significant symptom reductions and sustained user engagement. Together, these landmark works chart a clear evolution: from demonstrating the basic feasibility of mobile CBT to rigorously quantifying its efficacy, diagnosing engagement bottlenecks, and prescribing evidence-driven design standards. Going forward, future research should prioritize adaptive interventions, user engagement, and interdisciplinary collaboration to fully realize the promise of CBT-based mobile applications for global mental health.

Incorporating the clinical trial analysis from PubMed has provided essential insights into emerging trends within CBT-based mobile applications for treating depression. The current clinical landscape highlights a notable expansion of therapeutic strategies beyond traditional CBT, integrating novel modalities such as interpersonal therapy and mindfulness practices. The adoption of these therapies reflects a growing recognition of their complementary benefits—interpersonal therapy effectively addresses social and relational aspects contributing to depression (49, 50), whereas mindfulness techniques offer valuable tools for managing stress and enhancing emotional regulation (51). The emergence of these interdisciplinary therapeutic approaches signifies a critical shift toward holistic patient-centered care. Such integrative methodologies have demonstrated clear advantages, including improved patient engagement, greater flexibility, and potentially enhanced clinical outcomes due to their comprehensive approach to mental health management. Strengthening interdisciplinary and inter-institutional collaborations is another key factor facilitating this transformation. Enhanced collaboration enables pooling of diverse expertise, resources, and methodologies, thereby enriching research quality, increasing the generalizability of clinical trials, and accelerating innovation. Such collaborations, especially when extending internationally, can significantly amplify the impact and efficacy of clinical interventions.

The emphasis on rigorous research methodologies, particularly RCT and factorial experimental trials, underscores the commitment of the research community to uphold scientific robustness and reliability. RCT, recognized as the gold standard in clinical research, minimize biases and provide highly credible evidence, critical for validating mobile mental health interventions. Reviewing the selected clinical trials further emphasizes these points. Dixit et al. (52) demonstrated the effectiveness of digital interventions integrating multiple therapies, like CBT, interpersonal therapy, behavioral activation therapy, and dialectical behavior therapy, for postpartum depression, showing significantly greater improvement compared to controls. Similarly, Schuffelen et al. (53) found that digital CBT for insomnia substantially alleviated depressive symptoms in patients with comorbid insomnia and depression, highlighting the potential transdiagnostic applications of mobile interventions. Rothman et al.'s (54) study on digital therapeutics (CT-152) provided evidence for the effectiveness of digital CBT adjunctive to pharmacological treatment, further reinforcing the viability and complementary potential of digital mental health interventions.

Additionally, Weintraub et al. (55) highlighted the significant improvements in psychosocial functioning and depressive symptoms in adolescents with severe mood and psychotic spectrum disorders through app-enhanced CBT, advocating the benefits of integrating mobile technology to boost therapeutic adherence and engagement. Chen et al. (56) demonstrated that digital CBT for insomnia effectively prevented the onset of major depressive disorder among youths, reinforcing the preventive potential of mobile mental health interventions. Lastly, Watkins et al. (57) confirmed the effectiveness of CBT-based self-help apps in reducing depression symptoms among young adults, underscoring their scalability and public health value. Overall, these clinical trials collectively illustrate a clear trajectory toward integrative, robust, and collaborative clinical research in mobile mental health interventions. Embracing this trajectory through continued interdisciplinary collaboration and rigorous methodological standards promises further advancements in digital therapeutics, ultimately enhancing accessibility, effectiveness, and patient outcomes in mental health care (58).

The PsycINFO analysis further refines the clinical implications of our bibliometric findings by highlighting how CBT based mobile interventions for depression are embedded within broader transdiagnostic frameworks. The prominence of social phobia as a high centrality keyword indicates that many digital CBT programs for depression are designed to address overlapping symptom clusters of social anxiety and mood disturbance rather than isolated diagnostic categories. Canton et al. (59) shows that cognitive behavior therapy provides the most enduring and clinically meaningful benefits for generalized social phobia, often outperforming pharmacotherapy in the maintenance of treatment gains and showing additional advantages when combined with assertive clinical management, which underlines the value of sustained behavioral and cognitive skill practice in real world social contexts. When the PsycINFO co-occurrence structure is viewed alongside this evidence, social phobia emerges not as a peripheral topic but as a clinically important bridge that links CBT based mobile tools for depression with the wider literature on social anxiety, comorbidity, and transdiagnostic mechanisms of change.

Equally noteworthy is the appearance of Chinese cultural groups as a distinct keyword cluster, which suggests that a growing share of CBT based mobile interventions for depression is being developed for, or empirically tested in, Chinese populations. Ng and Wong show that cognitive behavioral therapy yields overall medium effect sizes on anxiety, depression, caregiving stress, and addictive behaviors among Chinese clients, with culturally adapted interventions achieving stronger short-term effects than unadapted protocols and with both short term and longer-term benefits remaining in the medium range (60). These results support the view that CBT is broadly compatible with Chinese cultural values when it is delivered in a structured, directive, and problem solves format, and they underscore the importance of cultural tailoring at the level of language, examples, family roles, and help seeking norms. In our PsycINFO corpus, the clustering of Chinese cultural groups together with digital intervention related terms indicates that mobile CBT applications are already being localized for Chinese users, which has direct implications for the global scalability and equity of evidence based digital mental health services.

Given the persistent concerns surrounding the long-term maintenance of CBT effects and the central role of behavioral intervention, we integrated evidence from recent randomized trials and meta-analytic syntheses that explicitly examine relapse prevention and behavioral activation. Uphoff et al. (61) shows that behavioral activation is at least as effective as standard cognitive behavioral therapies and medication across a wide range of adult depression trials and may be more cost efficient, reinforcing the notion that focused behavioral strategies can match more complex cognitive packages in acute symptom reduction. Fava et al. (62) shows that adding cognitive behavioral treatment of residual symptoms, supplemented by lifestyle modification and well-being therapy, markedly reduces 2 years relapse rates in patients with recurrent depression after antidepressants are tapered, compared with clinical management alone, emphasizing the importance of targeting residual symptomatology rather than relying solely on maintenance pharmacotherapy. Soleimani et al. (63) shows that group based behavioral activation yields greater improvement in depressive symptoms than group cognitive therapy in university students with subsyndromal anxiety and depression while maintaining comparable gains in anxiety, stress, and functioning, which highlights the efficiency of behavioral activation as a transdiagnostic prevention strategy. Jacobson et al. (64) shows that the behavioral activation component on its own produce outcomes equivalent to the full cognitive behavioral treatment for depression, suggesting that activation and engagement with rewarding environmental contingencies are the primary therapeutic engines of change. Hollon et al. (65) shows that cognitive therapy confers an enduring protective effect against relapse that is at least as strong as continuation antidepressant medication in patients with moderate to severe depression, and that patients who discontinue medication relapse more frequently than those who complete a course of cognitive therapy and then stop treatment. Zhou et al. (66) shows that in a network meta-analysis of psychological relapse prevention strategies, cognitive behavioral therapy offers the longest, although not continuous, protection against recurrence, while behavioral activation and interpersonal psychotherapy demonstrate delayed but significant advantages over placebo and all structured psychological interventions outperform supportive counseling across most follow up points. Taken together, this body of evidence converges with our PsycINFO findings by indicating that the durability of depression treatment depends heavily on sustained behavioral engagement, systematic activation, and the management of residual symptoms, and it suggests that CBT based mobile applications grounded in behavioral activation principles, adapted for specific cultural groups, and sensitive to comorbid social anxiety may be particularly well suited to support long term recovery trajectories in everyday life settings.

Beyond CBT, which remains a first-line psychological treatment for depression, our findings align with a broader literature showing that mindfulness-based approaches have gained wide recognition for their effectiveness in treating mood and anxiety disorders. Mindfulness is commonly defined as the deliberate, present-moment awareness of internal and external experiences with a non-judgmental and accepting attitude, and has given rise to structured interventions such as Mindfulness-Based Stress Reduction (MBSR) and Mindfulness-Based Cognitive Therapy (MBCT) (67–69). Alkan et al. (70) shows that mindfulness-based interventions including MBCT and related third-wave cognitive-behavioral approaches produce significant reductions in depressive symptoms across a wide range of psychiatric diagnoses, supporting mindfulness as a robust, transdiagnostic target for mood improvement. Montero-Marin et al. (71) shows that in recurrent depression, MBCT combined with antidepressant tapering support not only prevents relapse but also exerts its effects through improvements in mindfulness skills, with particularly pronounced benefits among individuals with higher baseline severity. At the comparative level, Li et al. (72) shows that there is no meaningful difference between mindfulness-based interventions and CBT in reducing anxiety, depressive symptoms, and sleep problems, indicating that mindfulness-based programs can function as viable alternatives to CBT in routine care. Additional clinical trials further underscore the utility of mindfulness in depression-related contexts: Norouzi et al. (73) shows that combining MBSR with physical activity augments standard pharmacotherapy for major depressive disorder by yielding greater improvements in depressive symptoms, anxiety, perceived stress, and sleep quality, while Zhu et al. (74) shows that adjunctive MBCT for adolescents with major depressive disorder significantly reduces suicidal ideation, depressive symptomatology, and circulating interleukin-6 levels, suggesting both psychological and inflammatory pathways of benefit. Together, these data position mindfulness-based interventions as both mechanistically meaningful and clinically effective counterparts or complements to CBT in depression care.

With the rapid expansion of digital health, mindfulness-based interventions are increasingly delivered through internet platforms and mobile applications, extending their reach and scalability in ways that are highly relevant to CBT-based mobile interventions for depression (75, 76). Wang et al. (77) shows that electronic health–delivered MBCT (eMBCT) produces small but significant and sustained reductions in anxiety and depression across twelve randomized controlled trials, with greater baseline symptom severity predicting larger gains, thereby supporting eMBCT as a feasible and effective modality for adult populations. Hurwitz et al. (78) shows that a mindfulness-enhanced internet-based CBT program implemented in routine care yields medium-to-large effect size reductions in depression and psychological distress, demonstrating that integrating mindfulness content into iCBT can improve outcomes at scale despite modest completion rates. At the level of mobile health, Liu et al. (79) shows that a mindfulness- and social support–based mobile application for postpartum women significantly increases perceived social support and parenting self-efficacy while reducing postpartum depressive symptoms, and Al-Refae et al. (80) shows that a self-compassion and mindfulness-based cognitive smartphone intervention leads to moderate reductions in depressive symptoms alongside gains in self-compassion and emotion regulation. Wu et al. (81) shows that online MBSR for psychiatric healthcare workers reduces depression and anxiety symptoms, with decreases in emotional suppression mediating these effects, highlighting a specific change process that may be leveraged in other digitally delivered mindfulness programs. Collectively, these findings indicate that mindfulness-based interventions, whether delivered in person or via internet and mobile platforms, occupy a distinctive position within the broader ecosystem of digital therapeutics for depression. They not only offer outcomes that are broadly comparable to CBT but also bring unique strengths in targeting transdiagnostic processes such as emotion regulation, self-compassion, and acceptance, while digital formats enhance accessibility, support stepped-care models in primary and community settings, and may improve adherence by embedding practice into the flow of everyday life.

In the past 12 years research into mobile CBT for depression has evolved into a rich and dynamic field where digital interventions have demonstrated meaningful clinical benefits while prompting deeper attention to user engagement and personalization (82). Innovations in app design have moved beyond standalone programs to blended care models and immersive experiences, with emerging work harnessing virtual reality and adaptive algorithms to tailor support to individual needs (83, 84). A growing emphasis on preventive applications and youth-focused initiatives underscores a shift toward early intervention and broader mental health promotion (85). Sustaining this momentum will depend on rigorous evaluation of real-world effectiveness, equitable access across diverse populations, and continued synergy among clinicians, technologists, and implementation scientists to translate digital CBT into scalable, lasting solutions.

In sum, the contribution of this study lies in linking a comprehensive, WoSCC-based scientometric map with a targeted analysis of randomized clinical trials identified via PubMed and a complementary behavioral science–oriented corpus derived from PsycINFO, thereby connecting publication structures to clinical endpoints and to transdiagnostic, culturally grounded perspectives on how app-based CBT for depression is conceptualized and implemented. Together, these three databases clarify that mobile CBT is moving toward adjunctive use alongside medication and psychotherapy, mechanistic targets such as sleep and emotion regulation, prevention in at-risk groups, regulatory-grade evaluation in formal trials, and culturally adapted digital interventions that address comorbid social anxiety and the needs of specific populations such as Chinese cultural groups. By integrating coauthorship and keyword networks from WoSCC and PsycINFO with trial evidence from PubMed, the analysis offers a coherent and multidimensional picture of where the field is consolidating and where scalable impact is most likely. Some constraints remain: CiteSpace mapping does not replace a full systematic review, cross-database harmonization is not perfect, citation indicators lag behind publication, and the exclusive focus on English-language publications introduces an inevitable language bias; consequently, impactful research published in local languages may have been omitted, potentially underrepresenting regional research trends. These caveats do not alter the directional signals observed here but highlight the value of periodic updates that draw on additional bibliographic sources and trial registries, expand non-English coverage, and include sensitivity checks to enhance reproducibility.

## 7 Conclusions

This bibliometric analysis systematically evaluated the global research trends and clinical progress in CBT-based mobile applications for depression treatment, incorporating literature from the Web of Science Core Collection, clinical trials from PubMed, and a complementary behavioral science–oriented corpus from PsycINFO. Results indicate rapid growth and increasing interdisciplinary collaboration, particularly within prominent institutions in the United States and Europe. The rising emphasis on randomized controlled trials underscores a commitment to methodological rigor and scientific validation within this research domain. Emerging clinical trends reflect a significant expansion beyond traditional CBT approaches, integrating complementary therapies such as interpersonal therapy and mindfulness practices. Accumulating randomized trials and meta-analytic evidence indicate that structured mindfulness-based interventions, including MBSR and MBCT, achieve reductions in depressive symptoms that are broadly comparable to CBT, contribute to relapse prevention in recurrent depression, and can also lessen suicidal ideation and stress-related biological markers in high-risk populations. These holistic methodologies offer substantial benefits, including enhanced patient engagement and emotional regulation. In parallel, internet-delivered and mobile application–based mindfulness programs, as well as mindfulness-enhanced digital CBT protocols, have demonstrated small to moderate but clinically meaningful improvements in depressive and anxiety symptoms across diverse groups, while extending access to care, embedding practice in daily life, and generating real-time data that can inform further refinement of intervention content and delivery. The adoption of advanced technological solutions, including adaptive algorithms, virtual reality, and blended care models, further highlights the evolving nature of mobile mental health interventions. Future research directions should focus on strengthening global interdisciplinary and inter-institutional collaborations to ensure diverse perspectives and enhance the generalizability of findings. Continued development and rigorous evaluation of personalized, adaptive digital interventions and preventive strategies are crucial. Emphasis should also be placed on improving user engagement, refining intervention designs, and exploring innovative technologies to optimize treatment efficacy and accessibility for diverse populations. Looking ahead to the next five to ten years, one particularly promising research stream is the integration of Artificial Intelligence (AI) and large language models (LLMs) into mobile CBT-based interventions for depression. Embedding AI-driven conversational agents and decision-support systems within mobile applications can enable more precise tailoring of therapeutic content, continuous analysis of user feedback, and dynamic adjustment of intervention components based on real-time symptom trajectories and engagement patterns. Such systems have the potential to enhance treatment adherence by providing on-demand psychoeducation, emotional support, and guided skills practice in users' everyday environments, while also generating high-resolution data that can be used to refine intervention mechanisms and optimize long-term outcomes. In addition, AI-enhanced mobile CBT interventions may extend beyond clinical populations to support prevention and early intervention in the general public by identifying at-risk individuals, delivering just-in-time adaptive support, and integrating with stepped-care models across primary care and community settings. Other emerging research streams are likely to include the convergence of mobile CBT with sensor-based digital phenotyping, wearable devices, and extended reality technologies to create more context-aware, immersive, and ecologically valid therapeutic experiences, as well as the development of culturally adaptive, multilingual platforms that can narrow global mental health disparities. Such comprehensive efforts promise significant advancements in digital therapeutics, ultimately improving mental health outcomes worldwide.

## Data Availability

The original contributions presented in the study are included in the article/supplementary material, further inquiries can be directed to the corresponding authors.
